# Case Ascertainment and Exposure Classification in Acute Myocardial Infarction Mortality Studies Involving Psychoactive Substance Use

**DOI:** 10.1002/clc.70202

**Published:** 2025-08-30

**Authors:** Noor‐ul‐Eman Haider, Syed Muhammed Rayyan, Muhammad Abbas

**Affiliations:** ^1^ Khyber Medical College Peshawar Pakistan; ^2^ Ayub Medical College Abbottabad Pakistan

Dear Editor,

1

We welcome the recent analysis of acute myocardial infarction (AMI) death in US adults aged ≥ 65 with documented psychoactive substance abuse disorders [1]. Attention by the authors to an elderly population is well‐timed, in light of increased co‐occurrence of cardiovascular disease and substance abuse in the elderly. Two important limitations—one clinical, the other methodological—are acknowledged, however, because they affect substantially both validity and interpretability of the results.

Epidemiological studies that use death certificate data for employment are fraught with a very high potential for case ascertainment bias in this particular situation. In elderly populations, AMI is frequently listed on death certificates by clinical history or circumstantial information in place of verifying electrocardiography or cardiac biomarker tests [2]. Challenging presentations, cross‐competing comorbidities, and silent myocardial infarction are not uncommon in this group and make both under‐ and overattribution of AMI as a cause of death more likely [3].

Equally problematic is the determination of psychoactive substance use. Toxicology is not the norm for older deaths unless there is obvious suspicion, and when it is done, it can be a restricted panel only [4]. This results in severe underreporting of drug use. Social stigma, clinician refusal to report, and ignorance regarding how to distinguish from prescribed versus nonprescribed use contribute to the risk of misclassification. When both outcome and exposure are tainted by classification error, cause‐specific mortality rate estimates can distort changes in reporting patterns rather than capturing epidemiologic trends. Follow‐up analyses using mortality registry data here need to be controlled for this dual‐source bias to maintain interpretive validity.

Second, operationalization of exposure—reducing all ICD‐10 F10–F19 categories into a single combined “psychoactive substance use disorder” category—is clinically restrictive. All of those codes cannot be collapsed into one usefully homogeneous category from the viewpoint of cardiovascular pathophysiology. Risk of AMI due to alcohol is secondary to chronic remodeling and arrhythmogenesis of the myocardium; cocaine and amphetamines via acute coronary vasospasm and proarrhythmic effects; opioids via hypoxia and bradyarrhythmia; sedatives via induction of hypotension and delay in conduction [5].

By combining these mechanistically different exposures, the study conceals substance‐specific patterns of mortality and prevents investigators from being able to determine whether trends are a result of stimulant‐induced acute events, alcohol‐induced chronic damage, or polypharmacy effects. This limitation reduces the value of the study for cardiologists, addiction specialists, and policymakers who need substance‐specific data to implement effective interventions. Further, the omission of poly‐substance interaction analysis may conceal combinations at high risk—such as co‐consumption of alcohol and benzodiazepines—that are synergistically fatal [6].

Generally speaking, these failures invalidate internal validity and clinical utility of findings. Registry‐based studies of cardiovascular disease with attention to alcohol and drug use among the elderly need to use more stringent case confirmation methods—preferably via medical record abstraction or autopsy data—and substance‐specific categorization to allow results to be interpreted as feasible prevention and treatment interventions [7]. These changes will not only enhance causal inference but also the potential for application in future work in this crucial and expanding field of cardiovascular epidemiology.

## Author Contributions

All authors contributed equally to this study.

## Ethics Statement

Ethics approval is not applicable.

## Consent

Informed consent is not applicable.

## Conflicts of Interest

The authors declare no conflicts of interest.

## Data Availability

The authors have nothing to report.
